# Selenium-enriched *Bacillus subtilis yb-1*14246 improved growth and immunity of broiler chickens through modified ileal bacterial composition

**DOI:** 10.1038/s41598-021-00699-4

**Published:** 2021-11-04

**Authors:** Jiajun Yang, Jing Wang, Kehe Huang, Qingxin Liu, GuofangLiu, Xiaozhou Xu, Hao Zhang, Mengling Zhu

**Affiliations:** 1 School of Animal Husbandry and Veterinary Medicine, Jiangsu Vocational College of Agriculture and Forestry, Jurong, 212400 Jiangsu China; 2 College of Animal Science and Technology, Chinese Agricultural University, Beijing, 100093 China; 3 College of Veterinary Medicine, Nanjing Agricultural University, Nanjing, 210095 China

**Keywords:** Applied microbiology, Nutrigenomics

## Abstract

Here, a Selenium-enriched *Bacillus subtilis* (SEBS) strain was generated and supplemented to broiler chickens’ diet, and the impact in ileum bacterial microbiome, immunity and body weight were assessed. In a nutshell, five hundred 1-old old chicken were randomly divided into five groups: control, inorganic Se, *Bacillus subtilis* (*B. subtilis*), SEBS, and antibiotic, and colonization with *B. subtilis* and SEBS in the gastrointestinal tract (GIT) were measured by fluorescence in situ hybridization (FISH) assay and quantitative real-time polymerase chain reaction (qPCR). In summary, Chicks fed SEBS or *B. subtilis* had higher body weight than the control chicks or those given inorganic Se. SEBS colonized in distal segments of the ileum improved bacterial diversity, reduced the endogenous pathogen burden and increased the number of *Lactobacillus* sp. in the ileal mucous membrane. Species of unclassified *Lachnospiraceae*, uncultured *Anaerosporobacter*, *Peptococcus*, *Lactobacillus salivarius*, and *Ruminococcaceae_UCG-014*, and unclassified *Butyricicoccus* in the ileal mucous membrane played a key role in promoting immunity. Inorganic Se supplementation also improved bacterial composition of ileal mucous membranes, but to a less extent. In conclusion, SEBS improved performance and immunity of broiler chickens through colonization and modulation of the ileal mucous membrane microbiome.

## Introduction

Probiotics are live microorganisms and spores, with beneficial health impacts in the body at certain doses^1^. Certain strains of *Bacillus subtilis*, when administrated orally, can colonize the intestinal mucous membrane, and optimize the bacterial composition, effectively stimulate immunity and metabolism^2^. These can play roles overcoming infection, stress and pathogens clearance^3,4^.

*Bacillus subtilis yb-1*14,246 strain was previously isolated from the ileum of a chick, in which its probiotic effects improved growth performance of the chickens^5^. Similar improvement was observed in the production performance of old laying hens upon dietary supplementation with *B. subtilis yb-1*14,246 strain^6^. Mechanistically, *B. subtilis yb-1*14,246 probiotic impacts are likely related to secretion of digestive enzymes namely protease, lipase, and amylase to digest the digesta in intestine^6^. The colonization of probiotic bacteria in gastrointestinal tract (GIT) is a crucial factor to play its role in interplaying with host^7,8^. Once the probiotic bacteria can colonize in the site of intestinal mucous membrane. They make use of the nutrition in GIT to propagate and secret the digestive enzymes to help the digestion of host^3,9^. This interaction between the probiotic bacteria and host was stronger than no colonized action of probiotic bacteria. Therefore, evaluation the colonization of *B. subtilis yb-1*14,246 is essential to make sure its approach of action^10^. Thereafter, to ensure the colonization of *B. subtilis yb-1*14,246 on the mucous membrane of ileum, further changed the ileal mucous bacterial composition and led to improved innate immunity and body growth is urgent.

Selenium (Se) is an important trace element with well-documented benefits^11^. Certain doses of Se supplementation can regulate metabolism and antioxidation^12^. Further, Se can help immune cells to defend against infection-causing pathogens^13,14^. Moreover, supplementation with se can modulate the bacterial composition in GIT, induced to good body health^15,16^.

Considering the beneficial effects of administration of Se and *B. subtilis yb-1*14,246 on bodily health^17,18^, we hypothesize that Se and *B. subtilis yb-1*14,246 in combination might led to additive or synergetic benefits. Therefore, we generated a Se-enriched *B. subtilis* (SEBS), which combines the benefits of *B. subtilis yb-1*14,246 and those of organic Se. This study could provide novel insights into the combined use of probiotic bacteria and the essential micro-element Se.

## Materials and methods

### Preparation and analyses of Bacillus subtilis yb-114246 and Selenium-enriched Bacillus subtilis yb-114246

*Bacillus subtilis yb-1*14,246 (BS) was isolated from the ileum of a healthy Chinese Huainan Partridge chicken by our research group at the institute of animal husbandry and veterinary medicine^6^, Anhui Academy of Agricultural Sciences. And stored at the China General Microbiological Culture Collection Center (CGMCC), the strain number is CGMCC 14246. The 16S ribosomal DNA was sequenced and deposited at the National Center for Biotechnology Information (NCBI) of the United States of America (USA) under the access number KT260179. *B. subtilis yb-1*14,246 was cultured in liquid beef extract peptone medium^6^. The fermentation of selenium-enriched *B. subtilis yb-1*14,246 (SEBS) was performed with sodium selenite supplemented into the culture medium. The morphological and structural properties of *B. subtilis yb-1*14,246 and SEBS were monitored with a scanning electron microscopy (SEM) and a transmission electron microscopy (TEM). *B. subtilis yb-1*14,246 and SEBS were concentrated via centrifugation at 3, 000 rpm, and immersed in a 5% glutaraldehyde solution for 24 h^19^. Se concentration in the supernatant and precipitate of *B. subtilis yb-1*14,246 and SEBS fermented medium was calculated by atomic absorption spectrometry, and the live bacteria were enumerated by colony forming units (CFU) in yeast extract peptone dextrose medium after ten times serial dilution^5^. The volume of 100 μL 10^6^ dilution was spread in a plate containing yeast extract peptone dextrose agar medium evenly. Then, the plate was laid in 37 °C for 16 h. The colonized number was enumerated to measure the CFU.

### Experimental design, birds, and diets

A total of 500 one-day-old Cobb broilers (average body weight, 40.05 g) were randomly allocated to five groups with five replicates of 20 each. Chickens were allowed ad libitum access to water and feed throughout the experimental period, and normal immunization program was implemented throughout the trial^20^. Chickens in the control group were fed a basal diet and the four treatment groups were fed the following: basal diet with either inorganic sodium selenite (IS), *B. subtilis yb-1*14,246 (BS), Se-enriched *B. subtilis yb-1*14,246 (SEBS), and flavomycin. Experimental diets were fed in two periods: starter (days 0–21) and finisher (days 22–42)^20^. The basal diet composition, which did not include any probiotics or antibiotics, and nutrient analysis results, are shown in Table 1. All nutrients met or exceeded the nutrient requirements of the national research council (NRC, 2012)^21^. For chickens in the IS group, 1.12 g of sodium selenite (analytically pure) was diluted in 100 mL distilled water and blended with 5 kg of basal feed. Thereafter, the mixed basal feed was added to a blender containing 95 kg of basal feed. The blender was employed for 20 min to ensure uniform mixing of additives. The feed for the flavomycin group was prepared using 4 g premixed food containing 10% flavomycin, which was blended with 100 kg of feed, to reach a concentration of 4 mg/kg. For the *Bacillus* group, 50 mL of *B. subtilis yb-1*14,246 fermentation liquid was measured separately and first blended with 5 kg of feed, and then with 95 kg of mass feed. The SEBS feed was prepared by blending 1000 mL of SEBS fermentation liquid with 100 kg of feed. After preparing the five different feedstuffs, the population of *B. subtilis yb-1*14,246 was counted using the plate method with a yeast extract peptone dextrose medium^5^. The concentration of Se in all feed types was also measured. The results are listed in Table 2.

**Table 1:** Nutrient analysis of the basal diet.

	Ingredient	Starter (0–21) %	Finisher (21–42) %
Item	Corn	58.12	61.75
	Soybean meal	29.15	26.45
	Fish meal	5.00	3.51
	Soybean oil	2.00	3.00
	Premix^a^	5.00^a^	5.00^a^
	Dicalcium phosphorus	0.47	0.29
	Limestone	0.26	0
Calculated nutrient	Metabolizable energy (MJ/kg)	12.02	12.49
	CP	21	17.5
	Calcium	1	0.85
	Total phosphate	0.68	0.65
	Available phosphorus	0. 5	0.42
	Lys	1.2	1.0
	Met	0.46	0.32

The premix provides, ^a^Vitamins and trace elements per kg diet: Vitamin A (retinyl acetate) 9, 875 IU, Vitamin D_3_ (cholecalciferol) 3, 000 IU, Vitamin E (dl-ɑ-tocopheryl acetate) 20 IU, menadione 3.25 mg, Vitamin B_12_ (cyanocobalamin) 0.025 mg, thiamin 1.5 mg, riboflavin 5.0 mg, biotin 0.032 mg, folacin 1.25 mg, niacin 12 mg, pantothenic acid 12 mg, and pyridoxine 3.75 mg, manganese 100 mg, zinc 80 mg, iron 80 mg, copper 8 mg, iodine 0.15 mg, and selenium 0.15 mg.

**Table 2:** Concentration of selenium and number of *Bacillus subtilis yb-1*14,246 in Groups.

Groups	Concentration of Se (ng/g)	Number of *Bacillus subtilis yb-1*14,246 (CFU/g)
Control (C1)	102.0	0
IS (C2)	602.0	0
BS (C3)	102.0	4.0 × 10^6^
SECB (C4)	602.0	4.0 × 10^6^
Flavomycin (C5)	102	0

Inorganic sodium selenite (IS), *Bacillus subtilis yb-1*14,246 (BS), Selenium-enriched *Bacillus subtilis yb-1*14,246 (SEBS).

### Performance and sample collection

Chicks in every replicate of each treatment group were weighed on days 0 and 42^20^. Daily feed consumption was accurately recorded. After 42 days, 2 chickens with an average body weight in each replicate were selected (n = 5 × 2), fasted for 12 h, and then the tissue were harvested under general halothane anesthesia. Ileum samples were removed under aseptic conditions, stored in sterile plastic tubes on ice, and immediately transported to our laboratory for quantification of assays.

### Fluorescence in situ hybridization (FISH) assay

The strain of *B. subtilis* residing in the GIT were investigated using FISH^15^. The probe was designed based on the 16S ribosomal ribonucleic acid of *B. subtilis* yb-1114246^22^, with sufficient length to ensure specific binding. Ileal mucosal samples (0.3 g) were fixed by immersion in 10% formaldehyde for 24 h. 50-μL of ileal mucosal homogenate was transferred to poly-l-lysine-coated slides and then air-dried on a sterile benchtop for 3 h. The tissue was then incubated with lysozyme at 32 °C for 10 min; washed with distilled water and immersed in 70% ethanol for 2 min, followed by air drying. Probes with carboxytetramethylrhodamine (sequence listed in Table 3) were designed and conjugated with deoxyribonucleic acid (DNA) of *B. subtilis yb-1*14,246. The probe was diluted to 60 nM, denatured at 95 °C for 5 min, and maintained at 4 °C before use. 12 μL of probe were then added to the tissue, followed by an incubation at 46 °C for 12 h, and washed with phosphate buffer solution (pH 7.4). The tissue was stained with 4′,6-diamidino-2-phenylindole for 5 min, then washed three times with distilled water for 5 min each. After drying, the slides were mounted with fluoromount-GTM (Abcam, Cambridge, UK) and observed with a fluorescence microscope (BX53; Olympus, Tokyo, Japan).

**Table 3:** The probes *Bacillus subtilis yb-1*14,246.

Bacteria	Genetic sequences 5′–3′
*Bacillus subtilis yb-1*14,246	CGCGATGTAGAGACTGATCGGCCACAATGGAACTGAGACACGGTCCATACTCCTACGTGAGGCTGCAGTAGGGAATCTTCCACAATGGTGCTCAAGCCTGATGCGAGCAACACCGCGTGAGTGAGAGAAGGGTTCGGCTCGTAAAGCT CTGTGTGTTGGAGAAGAACGTGGTGAGAGTAACTGTTCAGCAGTGACGGTATCCAGACCAGAAAGTCACGGCTAACTTACGTGCCAGCAGCCGCGG

### Quantitative real-time polymerase chain reaction for colonization of B. subtilis

After fermentation in beef extract peptone medium, a tenfold dilution series of *B. subtilis yb-1*14,246 was plated^18^. Colony forming units of *B. subtilis yb-1*14,246 were counted using the plate method under a microscope to obtain samples of 1 × 10^4^, 10^5^, and 10^6^. Total RNA in each dilution was extracted using the RNA Extraction Kit (Invitrogen, Carlsbad, CA, USA)^22^. Reverse transcription was performed using a GoScript Reverse System (Invitrogen). First-strand cDNA was synthesized by incubating a reaction mixture containing 11 μL RNA and 1 μL RNase-free dH2O at 70 °C for 3 min, followed by 0 °C for 5 min. A dNTP mixture (1 μL; 10 mmol/L), 4 μL GoScript 5X reaction buffer, 1 μL GoScript reverse transcriptase, 1.5 μL Mg^2+^ (25 mM), and 0.5 μL RNase inhibitor were combined in a total volume of 20 μL and incubated in a 37 °C in a water bath. Primers were designed according to the 16S rRNA of *B. subtilis* KT260179 and are described in Table 4. Amplification was performed in a 20-μL mixture containing 10 μL of 2 × qPCR SYBR Premix Ex-Taq, 2 μL template cDNA, 0.5 μL each primer (10 μmol/L), and 7 μL PCR-grade water. The cycling protocol was as follows: 95 °C for 30 s, followed by 40 cycles of 95 °C for 5 s and 60 °C for 30 s, and one cycle for melting curve analysis, consisting of 95 °C for 60 s, 65 °C for 60 s, and 95 °C for 1 s. The amplification curve was generated based on the dilution of the standard curve of *B. subtilis yb-1*14,246. The standard curve of *B. subtilis yb-1*14,246 was described according to the results of qPCR.

**Table 4:** All of the PCR primers.

Gene name	Forward		Reverse
*Bacillus subtilis yb-1*14,246	ACATCCTCGAAGATACAGTGAGA		GCATGACAACTACCACGACCT
TNF-α	CCACAGCTCCGCTCAGAAC	GAGAGGACGATGCCACGAC	
IFN-γ	AACCTTCCTGATGGCGTGAA	AAACTCGGAGGATCCACCAG	

Tumor necrosis factor-α (TNF-α, interferon-β (IFN-β).

Samples (0.2 g) of mucous membrane from the distal segment of the ileum were prepared to extract total RNA and qPCR was conducted as described above to evaluate colonization of *B. subtilis*^23^.

### Gut bacterial 16S rDNA sequence and analysis

Samples (0.25 g) of the ileal mucous membrane were prepared. Ten replicates were prepared in each group (n = 5 × 10), and microbial DNA was extracted. Final DNA concentration and purity were determined using a NanoDrop 2000 UV–Vis spectrophotometer (Thermo Scientific, Waltham, MA, USA), and DNA quality was determined using 1% agarose gel electrophoresis^24^. The V3–V4 hypervariable regions of the bacterial 16S rRNA gene were amplified with primers *338F* (5′-ACTCCTACGGGAGGCAGCAG-3′) and *806R* (5′-GGACTACHVGGGTWTCTAAT-3′) using a thermocycler PCR system (GeneAmp 9700, Applied biosystems, Foster City, CA, USA). PCR was conducted as follows: 3 min of denaturation at 95 °C, 27 cycles: 30 s at 95 °C, 30 s of annealing at 55 °C, 45 s of elongation at 72 °C, and a final extension at 72 °C for 10 min. PCR was performed in triplicate in 20-μL mixtures containing 4 μL of 5 × FastPfu Buffer, 2 μL of 2.5 mM dNTPs, 0.8 μL of each primer (5 μM), 0.4 μL of FastPfu polymerase, and 10 ng of template DNA. The resulting PCR products were extracted from a 2% agarose gel and further purified using the AxyPrep DNA Gel Extraction Kit (Axygen Biosciences, Union City, CA, USA) and quantified using QuantiFluor™-ST (Promega, Madison, WI, USA) according to the manufacturer’s protocol. Purified amplicons were pooled in equimolar concentrations and paired-end sequencing was performed (2 × 300) on an Illumina MiSeq platform (Illumina, San Diego, CA, USA) according to standard protocols. The Illumina sequencing raw data have been deposited into the Sequence Read Archive database (SRP) of NCBI (SRR13290974). The BioProject accession number is PRJNA684959.

### qPCR assays for chicken ileal immune cytokines

Chicken mucosal tissues, collected from the distal segment of the ileum, were washed with ice-cold PBS to remove intestinal contents, and sectioned longitudinally into small specimens^23^. Chicken mucosal cells were isolated using a PBS buffer containing 1 mM EDTA, 1 mM dithiothreitol, and 5% fetal bovine serum, with shaking at a speed of 60 rpm/min at 37 °C for 10 min. Samples of cells were prepared to extract total RNA to evaluate the level of the immune cytokines of tumor necrosis factor-α (TNF-α) and interferon-β (IFN-β). Relative expression levels of target genes were quantitatively normalized against the expression of GAPDH using the ΔΔCT method. Primers for TNF-α and IFN-β were designed according to the chicken RNA genes submitted to NCBI. All PCR primers used in this study are described in Table 4.

### Statistical analyses

To facilitate statistical analysis, the names of C1, C2, C3, C4, and C5 were instead of control, SS, BS, SEBS and flavomycin groups respectively in all figures and tables. Body weight, Se concentration, qRT-PCR, and DNA sequencing data were subjected to one-way ANOVA using the GLM procedure of SPSS, with significance reported at *P* < 0.05. Means were further separated using Duncan’s multiple range test^6^. All data were statistically processed as repeated measures to determine the interaction of Se and *B. subtilis*. A *P* value of less than 0.05 was considered statistically significant.

Diversity metrics were calculated using the core-diversity plugin within QIIME2^24^. Feature level alpha diversity indices and operational taxonomic units (OTUs) were used to estimate the microbial diversity within an individual sample. Beta diversity distance measurements were performed with weighted UniFrac to investigate the structural variation in the microbial communities across samples, and then visualized via principal coordinate analysis (PCoA). Co-occurrence analysis between mRNA of immune cytokines of TNF-α, IFN-β and species of bacteria in ileal mucous membrane was performed by calculating Spearman’s rank correlations and the network plot. Additionally, the potential Kyoto Encyclopedia of Genes and Genomes (KEGG)^25^ Ortholog functional profiles of microbial communities were predicted using PICRUSt.

### Animal ethics statement

All study procedures were approved by the Animal Care and Use Committee of China Agricultural University and were in accordance with the Guidelines for Experimental Animals established by the Ministry of Science and Technology (Beijing, China). All efforts were obeyed the rules of animal welfare and were to minimize animal suffering. All the authors confirm that the study is reported in accordance with ARRIVE guidelines (https://arriveguidelines.org). 

## Results

### Analysis of SEBS

SEBS was acquired after 24 h of fermentation of *B. subtilis yb-1*14,246 in medium containing inorganic sodium selenite. The fermented medium color was pale pink. The contents of ionic Se in the supernatant and precipitate of SEBS were analyzed with hydride generation atomic absorption spectrometry (HG-AAS), and the contents were 1.77 μg/mL and 48.13 μg/mL, respectively. In the precipitate of SEBS fermentation, Se was existed primarily as Se protein (valence 2-) and nanoparticles of Se (valence 0) in the cells of *B. subtilis yb-1*14,246. The live cell of *B. subtilis yb-1*14,246 and SEBS both reached 9.2 × 10^8^ CFU/mL. Appearance and internal structure did not change between *B. subtilis yb-1*14,246 and SEBS, as proved by SEM and TEM.

### Impact on growth performance and mortality

**Figure 1:**
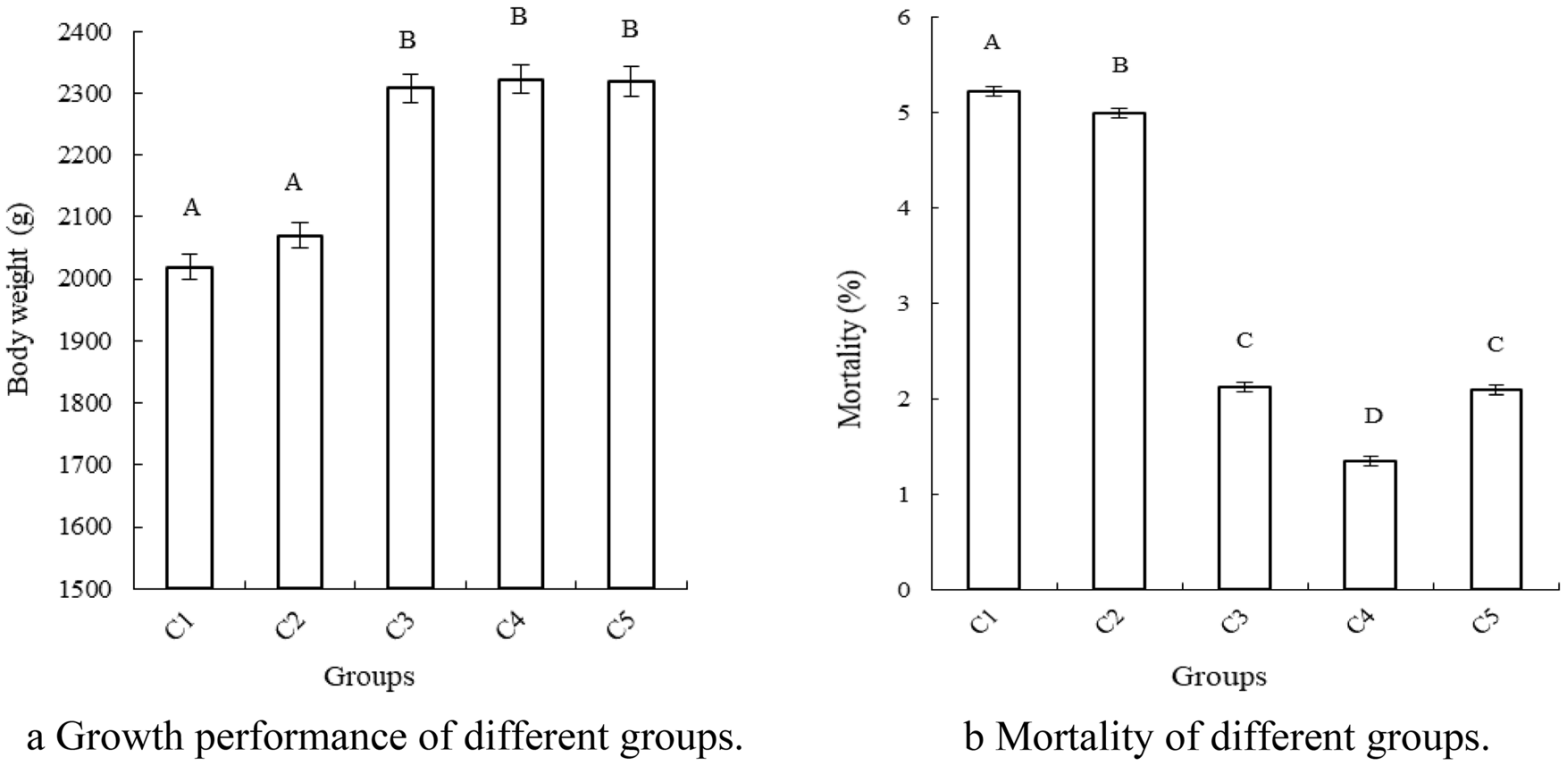
Chicken growth performance and mortality. Chicks fed control (C1), or with SS (C2), BS (C3), SEBS (C4) and flavomycin (C5) supplementation and growth performance (**a**) and mortality (**b**) of each group was measured. Data was statistically processed as repeated measurements. Superscript capital letters in columns mean P < 0.01. The Microsoft Office Excel 2010 was used to as the stamp software.

To distinguish the effects between *B. subtilis yb-1*14,246 and SEBS, the indexes of body weight of chicks and mortality of broiler chickens were calculated (Fig. 1). The final body weight of chicks administrated SEBS was significantly higher (*P* < 0.01) than those of the control and inorganic Se groups, with a body weight increase of 303 g (Fig. 1a). The mortality of chicks with *B. subtilis yb-1*14,246, SEBS, and flavomycin supplementation was significantly declined (Fig. 1b, *P*  < 0.01). The mortality under SEBS supplementation was the lowest, with a decrease of 3.87 compared to that in the controls (*P* < 0.01).

### Colonization and levels of B. subtilis yb-114,246 and SEBS in intestine

**Figure 2:**
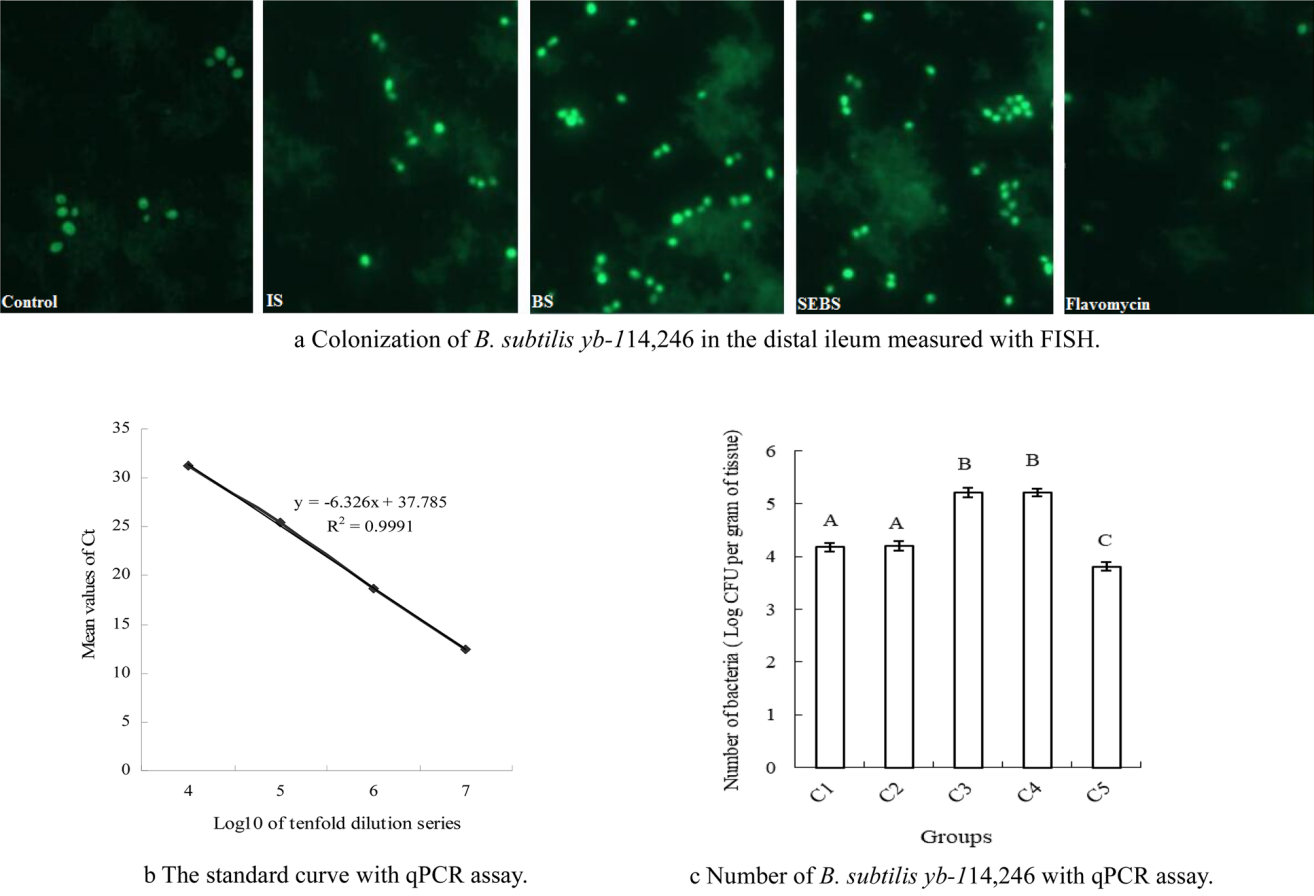
Colonization of *B. subtilis yb-1*14,246. (**a**) *B. subtilis yb-1*14,246 in the distal ileum was detected with FISH and (**b**) the total number of *B. subtilis yb-1*14,246 estimated based on a standard curve by qPCR assay (**c**). Values represented by vertical are means, with standard errors are represented by vertical bars. Significant differences from the control group were determined by one-way ANOVA followed by Duncan’s multiple comparison tests: superscript capital letters in columns mean P < 0.01. The Microsoft Office Excel 2010 was used to as the stamp software.

The colonization of *B. subtilis yb-1*14,246 in intestinal mucous membranes was measured by FISH and qRT-PCR. *B. subtilis yb-1*14,246 is indicated by green spots detected in the distal segment of the ileum by the FISH assay (Fig. 2a) for both *B. subtilis yb-1*14,246 and SEBS supplementation. The qRT-PCR assay results were consistent with the FISH results. The standard curve of *B. subtilis yb-1*14,246 was based on the tenfold dilution series of the fermented culture (Fig. 2b). *B. subtilis yb-1*14,246 growth increased in the distal segment of the ileum (Fig. 2c, *P* < 0.01).

### SEBS optimized ileal microbiota

**Figure 3:**
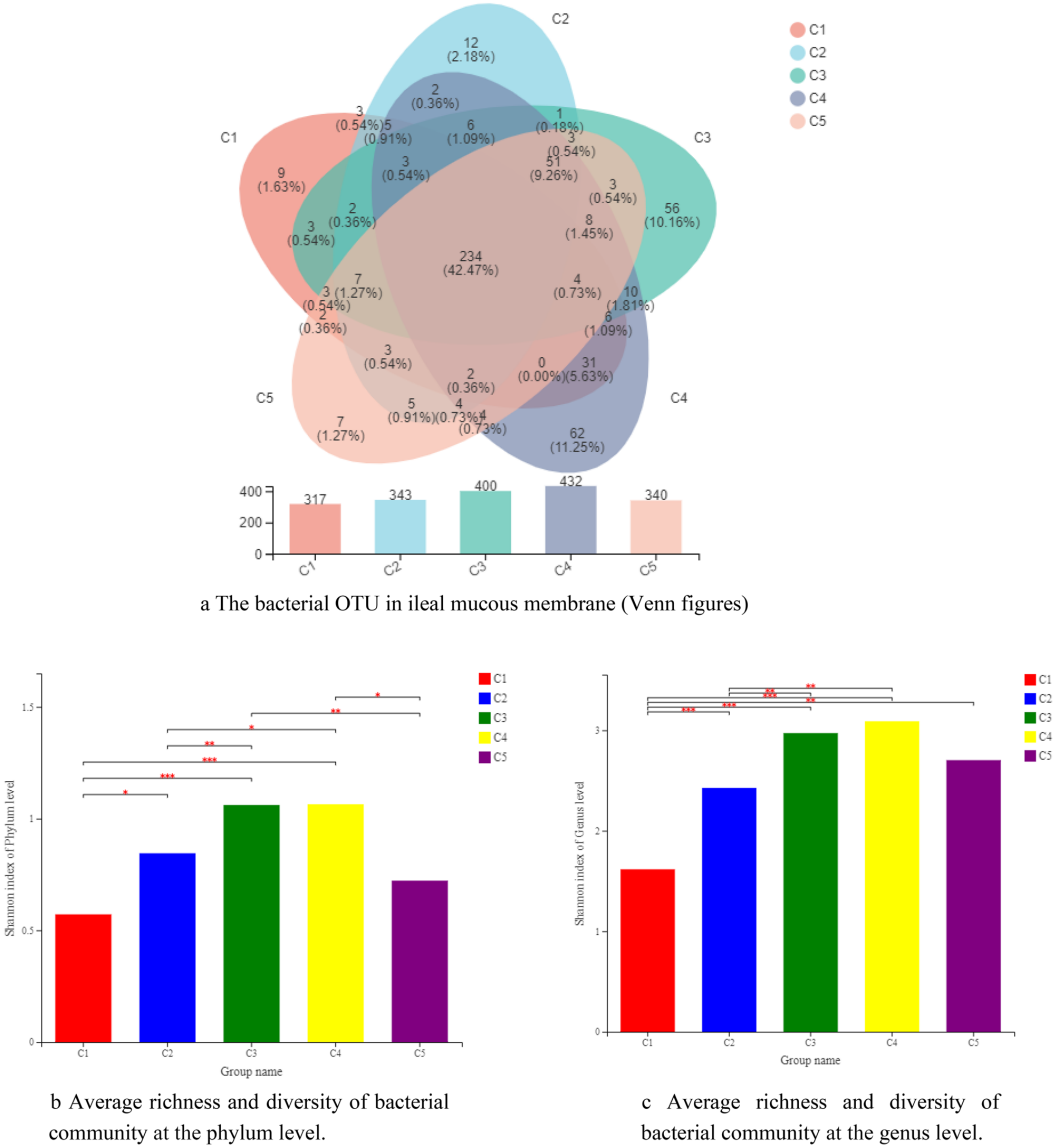

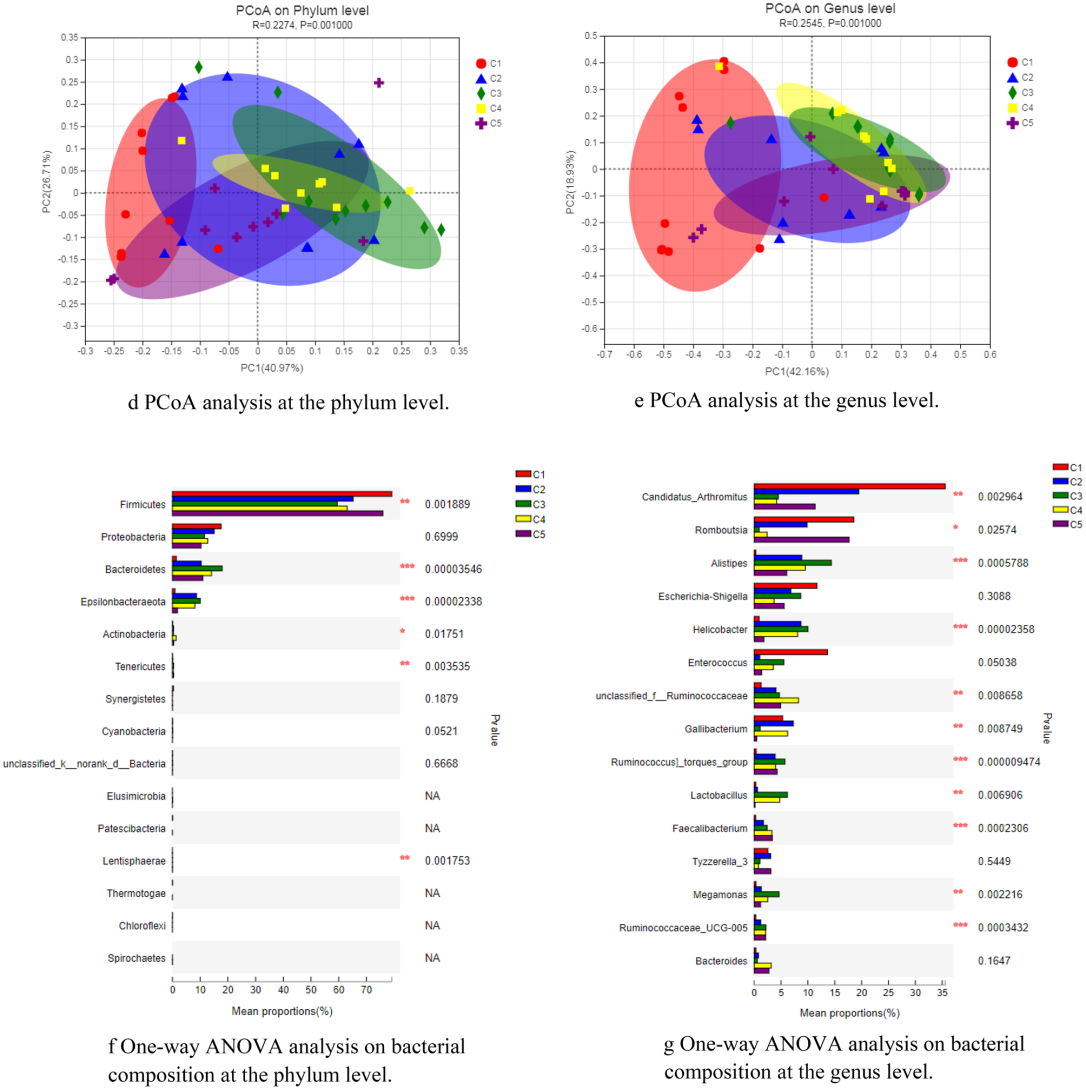
Overall profile of bacterial composition in ileal mucous membrane. (**a**) The bacterial OTU in ileal mucous membrane (Venn figures). The number in the color circus or overlapped circus represented the owned OTU in one or more groups and correlated proportion. (**b**) Bacterial composition at the phylum level. (**c**) Bacterial composition at the phylum genus level. *in the same column means P < 0.05, **P < 0.01, and ***P < 0.001. (**d**,**e**) PCoA analysis of UniFrac distance metric of bacterial OTUs at the genus level (**d**) and phylum level (**e**). Figure was drawn by the ANOSIM of UniFrac distance metric. (**f**,**g**) One-way ANOVA analysis of bacterial composition at the phylum level (**f**) and phylum genus level (**g**). * n the same column means P < 0.05, **P < 0.01, and ***P < 0.001. The Microsoft Office Excel 2010 was used to as the stamp software.

Changes of bacterial composition in ileal mucous membranes caused by *B. subtilis yb-1*14,246 colonization were detected by the next generation of sequencing technology was employed. High-throughput sequencing of all samples produced a total of 602,704 clean tags, which were identified as a total of 551 OTUs (Fig. 3). This sequencing depth closely reflects the total microbial species richness. The number of OTUs in control, IS, BS, SEBS and flavomycin was 317, 343, 400, 432, 340, respectively. Chicks with SEBS supplementation is the highest number. Bacterial composition in all supplementary groups was improved compared with control. All five groups are represented by 234 OTUs, contributing 42.47% of the total proportion. Bacterial composition in the ileum showed few differences between SEBS and BS supplementation through the alpha diversity (Shannon index) of mucous bacterial composition both in phylum and genus levels (Fig. 3b,c). The similarities of the weighted UniFrac-based PCoA indicated that the main factors caused 67.68% of the variations (Fig. 3d, *R* = 0.2274), which influenced the composition of the microbiota. Results of bacterial community structure indicated that the numbers of three specific phyla, namely *Bacteroidetes*, *Actinobacteria*, and *Epsilonbacteraeota*, significantly increased with *B. subtilis yb-1*14,246 and SEBS supplementation compared with those in controls, with the numbers of *Actinobacteria* being higher than those in the IS and flavomycin groups (Fig. 3e).

At the genus level, all supplemented groups were more abundant than those of the control (Fig. 3c,f). Six genera, namely *Candidatus Arthromitus*, *Romboutsia*, *Escherichia-Shigella*, *Enterococcus*, *Gallibacterium*, and *Tyzzerellawere*, represented 87.28% of the total proportion, with *Candidatus Arthromitus* covering 35.54% in control group (Fig. 3f). Further, the genera of *Alistipes*, *Helicobacter*, *Ruminococcaceae*, and *Ruminococcus* were detected in five supplementation groups, and *Lactobacillus* and *Bacteroides* were found in the *B. subtilis yb-1*14,246 and SEBS groups. Similarities of PCoA showed that two main factors influenced the bacterial cluster at the genus level of bacteria, indicating a ratio of 61.09% (Fig. 3e, *R* = 0.2545). In the PCoA of bacterial OTUs, the SEBS group, samples were homogenous (more clustered together), while the controls were the most heterogenous (scattered). These suggested that the bacterial communities were most stable and optimal with SEBS supplementation.

### SEBS improves immunity and metabolism

**Figure 4:**
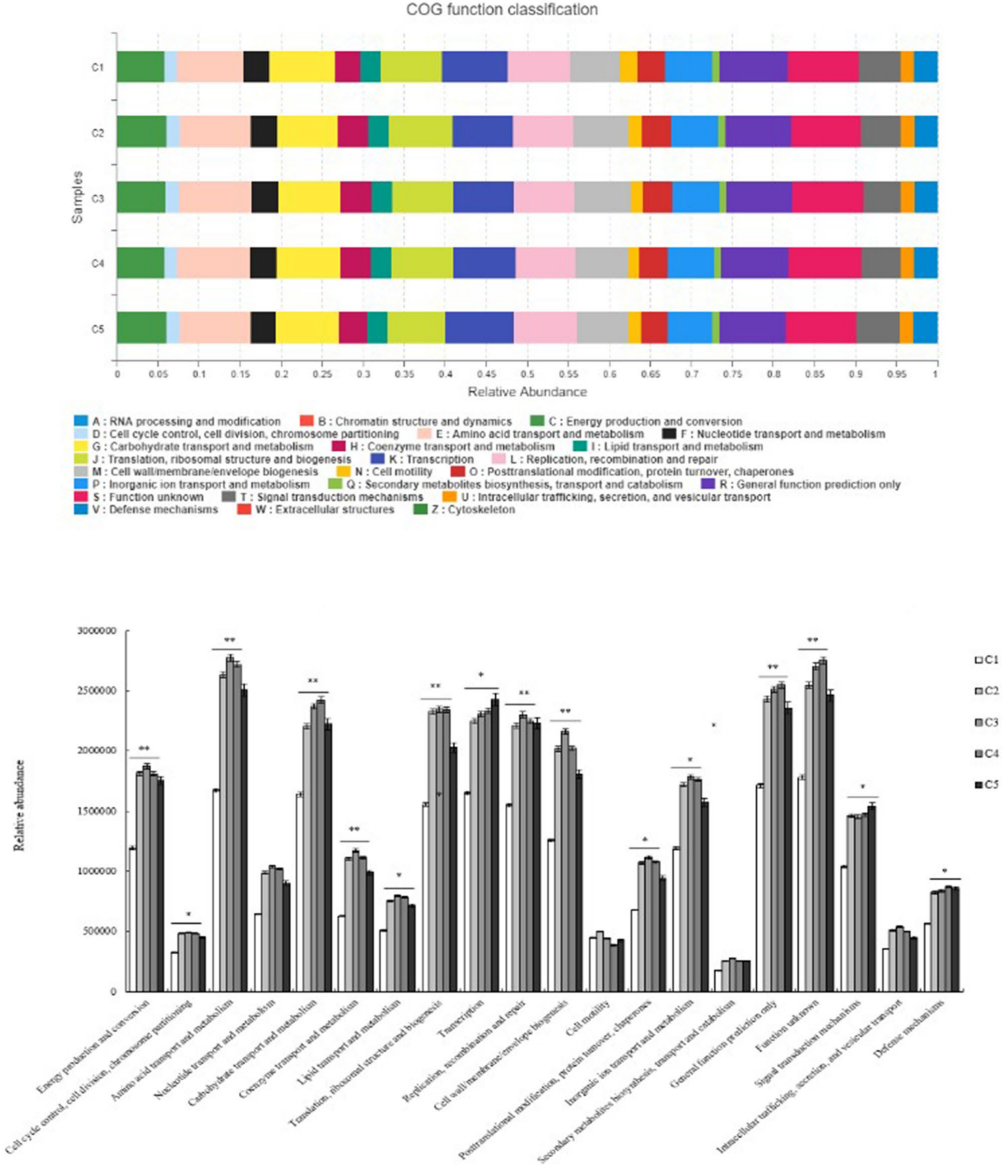

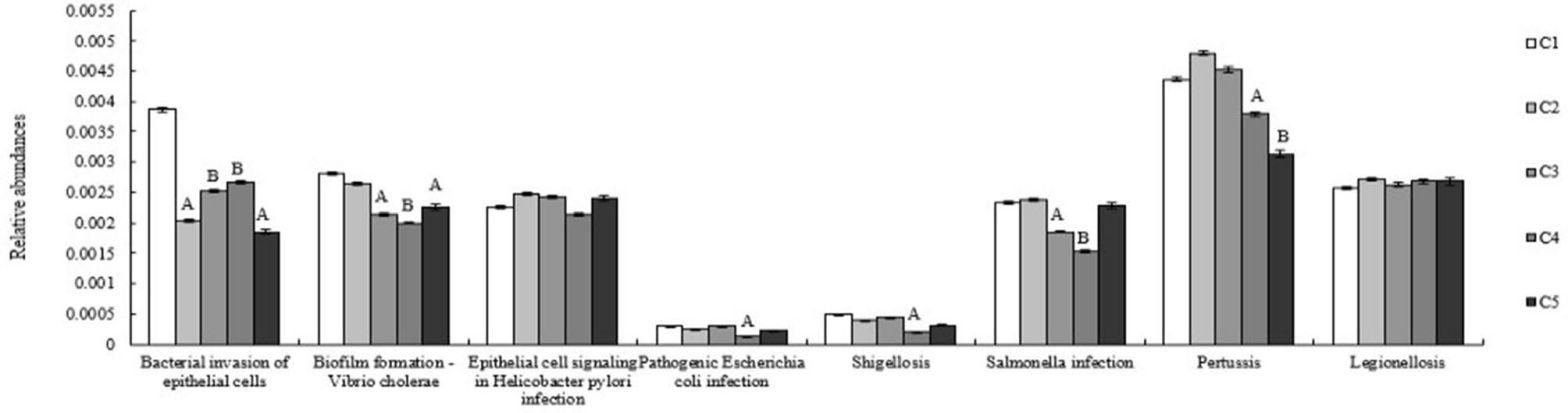
Function classification based on bacterial composition. (**a**) KEGG pathway function classification. KEGG is a database resource that integrates genomic, chemical and systemic functional information^25^. STAMP software was applied to detect the differentially abundant Kyoto Encyclopedia of Genes and Genomes (KEGG) pathways among groups with false discovery rate correction. (**b**) One-way ANOVA analysis between bacterial composition and function. *P < 0.05, **P < 0.01. (**c**) Relative abundance of pathogen in One-way of ANOVA analysis of KEGG pathway function classification caused by pathogenic bacteria. Superscript capital letters in the same color of column and in the same experimental duration mean P < 0.01. The one-way ANOVA analysis was performed by post hoc tests. The Microsoft Office Excel 2010 was used to as the stamp software.

The relationship between the microbiota of ileal mucous membranes and chicken body function was assessed through KEGG pathway classification and one-way ANOVA. 16SrDNA sequencing data from ileal mucous samples unveiled the metabolism and digestion of nutritional substances, DNA, RNA and protein expression influenced by bacterial OTU in phylum or genus levels. Dominate genus of bacterias were chosen to reflect this relationship between bacterial composition and function. These results (Fig. 4a,b) indicated that more microbes on diversity and number were found, contributing to more genetic expression of amino acids, carbohydrates, co-enzymes, lipid transport and metabolism (*P* < 0.01), energy production and conversion (*P* < 0.01), signal transduction mechanisms, and defense mechanisms (*P* < 0.05) to chicken. Further, primary infectious diseases caused by pathogenic bacteria were analyzed (Fig. 4c), which showed that all four supplements strengthened defenses against bacterial invasion of chicken epithelial cells, compared to controls (*P* < 0.01).

Significantly altered OTU on genus level in ileal mucous membrane were chosen to analyze the differences in pathogen burden according to the sequencing statistical results. We chose the pathogen covered major proportion and easily caused disease, such as bacterial invasion of epithelial cells, biofilm formation-Vibrio cholerae, epithelial cell signaling in Helicobacter pylori infection, pathogenic Escherichia coli infection, shigellosis, salmonella infection, pertussis and legionellosis. With *B. subtilis yb-1*14,246, SEBS, and flavomycin supplementation, body defenses against biofilm formation by *Vibrio cholerae* improved significantly (*P* < 0.01). Further, defense against *Salmonella* infection and Pertussis improved in two *B. subtilis yb-1*14,246 and flavomycin supplementation groups. Moreover, chicks receiving SEBS exhibited enhanced defense against pathogenic *Escherichia coli* infection and Shigellosis (*P* < 0.01) (Fig. 4c) according to one-way ANOVA analysis.

**Figure 5:**
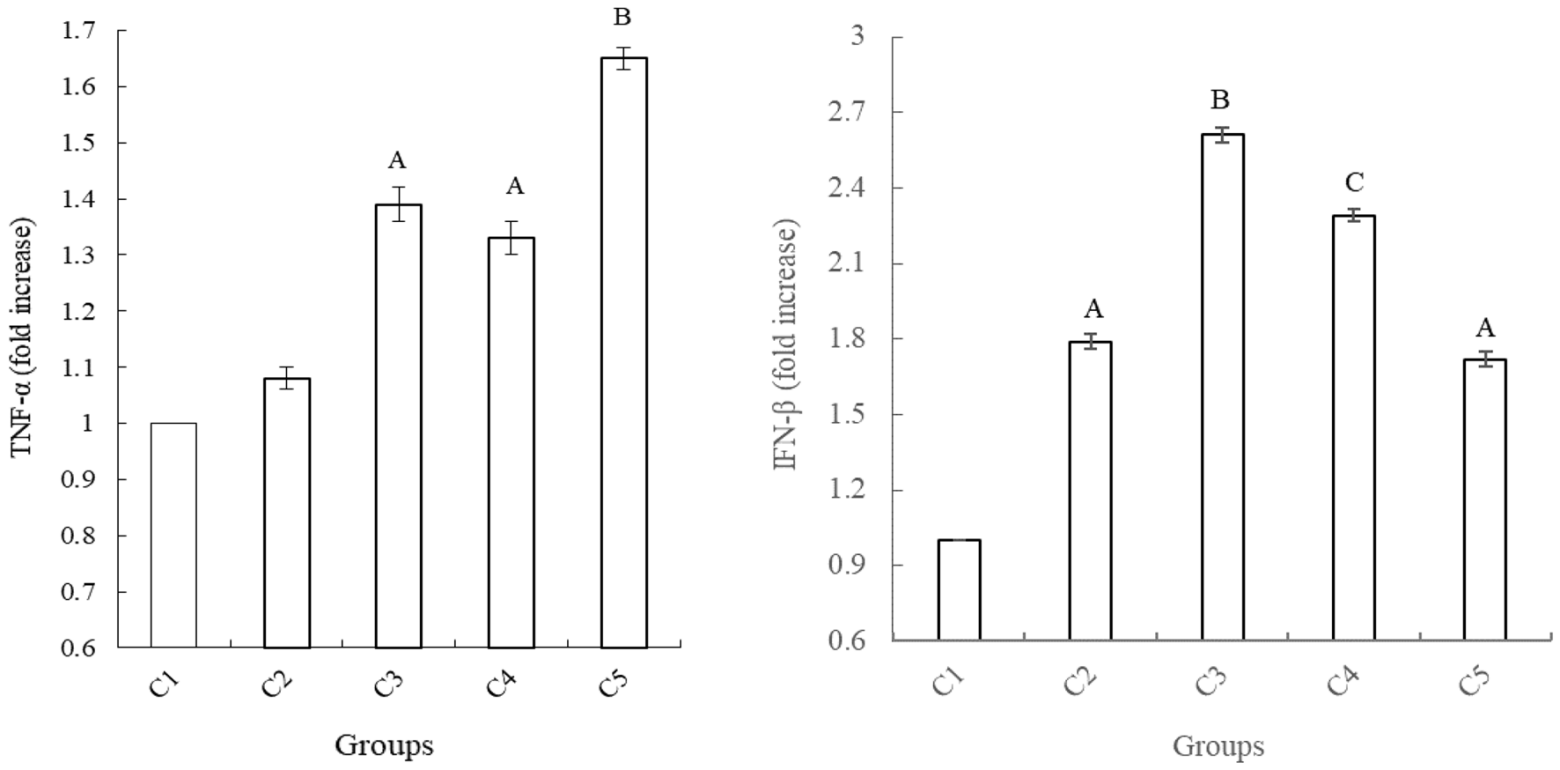
The expression of cytokines in intestinal mucous cells. mRNA: glyceraldehyde phosphate dehydrogenase (GAPDH) internal control was used for statistical comparison. Values represented by vertical bars are means with standard errors. Significant differences from the control group were determined by one-way ANOVA followed by Duncan’s multiple comparison tests: superscript capital letters in the same color of column and in the same experimental duration mean P < 0.01. The Microsoft Office Excel 2010 was used to as the stamp software.

TNF-α, IFN-β are two important anti-infection factors in body immunity, and were therefore chosen to evaluate the immune status. Immune cytokines TNF-α, IFN-β in the chicken ileal mucosa were further quantified using qRT-PCR. The mRNA expression of cytokines TNF-α and IFN-β were monitored (Fig. 5), with increased expression of these two cytokines observed in in SEBS, BS, and flavomycin groups, compared to controls (*P* < 0.01). In the IS-supplemented group, the expression IFN-β were significantly improved compared with control groups (*P* < 0.01). The expression of cytokines TNF-α and IFN-β in the *B. subtilis yb-1*14,246 group was higher than those observed in the SEBS group (*P* < 0.01).

### Relationship of bacterial composition with intestinal immunity

**Figure 6:**
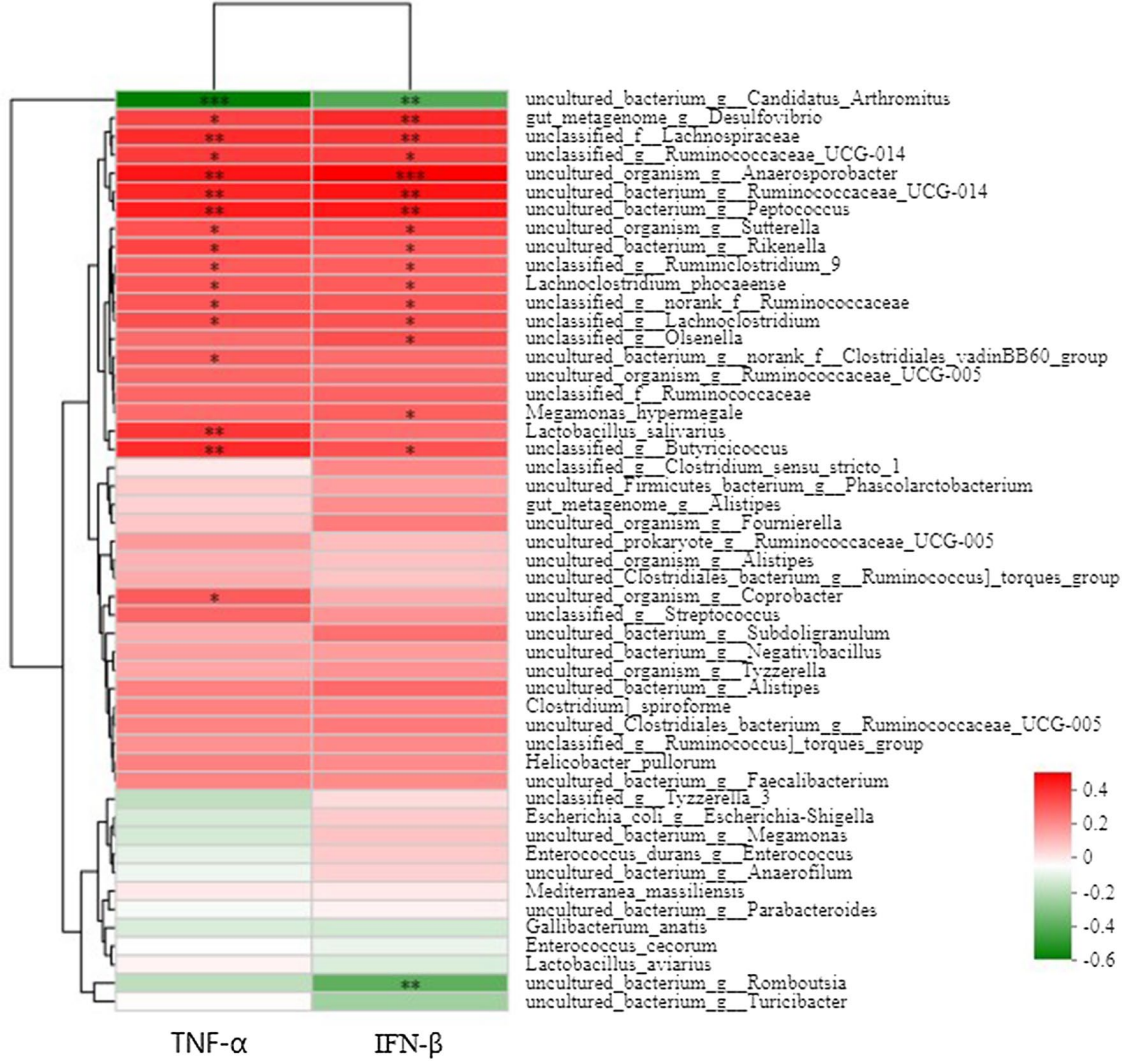
Spearman’s correlation analysis for TNF-α, IFN-β mRNA expression and species of bacteria in ileal mucous membrane. *P < 0.05, **P < 0.01, ***P < 0.001. Relative abundance is indicated by a color gradient from green to red, with green representing low abundance and red representing high abundance.

The relationship between certain bacterial species and TNF-α and IFN-β genetic expression was assessed by Spearman’s correlation analysis. The results (Fig. 6) showed that the abundance of uncultured *Candidatus arthromitus* and *Romboutsia* species were negatively correlated with mRNA genetic expression of TNF-α and IFN-β in ileal mucous membranes (*P* < 0.01), In contrast, abundancy of unclassified *Lachnospiraceae*, uncultured *Anaerosporobacter*, uncultured *Ruminococcaceae_UCG-014*, uncultured *Peptococcus*, *Lactobacillus salivarius*, and unclassified *Butyricicoccus* species were positively correlated with mRNA expression of TNF-α and IFN-β (*P* < 0.01). Finally, species of unclassified *Lachnospiraceae*, uncultured *Anaerosporobacter*, *Ruminococcaceae_UCG-014*, *Peptococcus*, *Lactobacillus salivarius*, and unclassified *Butyricicoccus* were increased in ileal mucous membranes of chick with SEBS supplementation (*P* < 0.01).

## Discussion

To explore the effects of combined use of Se and *B. subtilis yb-1*14,246. SEBS was cultured firstly, then its morphological and biochemical characteristics were analyzed. The characteristic of SEBS did not show any changes with *B. subtilis yb-1*14,246 after bio-transforming with inorganic Se. Selenomethionine was proved as the primary ionic form of Se in bacteria after bio-transformation^26^. The ionic form of Se in the supernatant and precipitate of fermented SEBS medium constituted nano-Se in red particles^27^. The composition of Se in the supernatant and precipitate of fermented SEBS medium was mainly in forms of selenomethionine and nano-Se particles, which conferred a pale pink color to the medium. The valences of Se changed from 4^+^ to 2^−^ and 0. Furthermore, broiler chickens with SEBS supplementation unveiled the effect on intestinal innate immune expression of BD1 and its potential mechanism.

Chicks with SEBS supplementation, *B. subtilis yb-1*14,246 and flavomycin showed higher final body weights, and this result was consistent with those of previous studies^28,29^. Both Se and *B. subtilis yb-1*14,246 can stimulate the growth of chickens^30,31^, however, chicks with IS supplementation showed no significant increases in final body weight. In our study, we supplemented basal feedstuff with a dose of 0.5 μg/g Se in an inorganic form, which had no inducing effects on broiler chickens, which was in accordance with our previous studies^32^.

Exploring the colonization of bacteria in vivo must be more eloquent than in cells in vitro*.* A previous study reported that the composition of ileum was most abundant among three segments of small intestine, with owing 10^7^ CFU/g bacteria^33^. Additionally, *B. subtilis* preferentially colonized in the ileal mucous membrane^34^. Our results also suggested that in chicks fed with *B. subtilis yb-1*14,246, these can colonize the ileal mucous membranes, as proved by FISH and qRT-PCR assays. The bacteria make use of nutrients in the intestine for propagation and paly beneficial roles on the chick, indicating a reciprocal relationship^35,36^. With growth of bacteria, metabolites of *B. subtilis yb-1*14,246 including antimicrobial substances and digestive enzymes, such as protease, lipase, and amylase, which maintained health and broke down feedstuff for nutrient absorption^37^. Chickens receiving SEBS had a higher final body weight with improved feed utilization efficiency than control chickens and the group receiving *B. subtilis yb-1*14,246 or Se alone, indicating an additive effect of these two supplements together.

Accompanied by the colonization of *B. subtilis yb-1*14,246, immunity was also improved through such reciprocal pathways. The colonization of *B. subtilis yb-1*14,246 in the ileal mucous membrane improved bacterial composition in the phylum and genus levels*.* The genus of *Lactobacillus*, *Peptococcus*, *Butyricicoccus*, and *Ruminococcaceae_UCG-014* with probiotic functions to body were enriched in chicks with supplemented with *B. subtilis yb-1*14,246 and SEBS^24,38,39^. Also, the proportion of conditioned pathogens or pathogens, namely *Escherichia-Shigella*, *Vibrio cholerae*, *Salmonella*, and *Pertussis bacilli*, significantly decreased, which led to improved immunity.

The species of *Lachnospiraceae*, *Ruminococcaceae_UCG-014*, *Peptococcus*, *Lactobacillus salivarius*, *Butyricicoccus,* and *Anaerosporobacter* can improve overall chicken health^40–43^. Abundant species of unclassified *Lachnospiraceae*, uncultured *Anaerosporobacter*, uncultured *Ruminococcaceae_UCG-014*, uncultured *Peptococcus*, *Lactobacillus salivarius*, and unclassified *Butyricicoccus* were identified in *B. subtilis yb-1*14,246 and SEBS supplemented groups. The species of *Lachnospiraceae*, *Ruminococcaceae_UCG-014*, *Peptococcus*, *Lactobacillus salivarius*, *Butyricicoccus,* and *Anaerosporobacter* improved overall health^40–43^. Moreover, *Lachnospiraceaer*, *Ruminococcaceae_UCG-014*, *Peptococcus*, *Lactobacillus salivarius*, and *Butyricicoccus* aid in digestion and nutrient absorption, whose abundances were all increased in SEBS-supplemented chicks, and KO functional profiles of microbial communities indicated that metabolism and immunity were improved by these species of bacteria. These results suggest that body immunity was improved by optimized intestinal microbiota in *B. subtilis yb-1*14,246 and SEBS supplemented chickens.

In addition, supplementation with 0.5 μg/mg Se in diet showed improved effects on bacterial composition in ileal membrane of chick. Our results advised that the bacterial abundances in phylum level were increased, with more OTUs in *Actinobacteria*. In further, the abundances in *Lactobacillus*, *Ruminococcaceae*, and *Ruminococcus* were also enriched with Se supplementation. These results were also reported in other studies, where selenium supplementation in nano particles benefited some genus of beneficial bacteria such as *Faecalibacterium prausnitzii* and *Lactobacillus* in poultry gut^44,45^. Se supplementation enriched the bacterial diversity compared to that in controls, which enhanced nutrient metabolism and immunity, as indicated by the results of KEGG function classification. Therefore, supplementary Se, at a suitable dose, could help body establish improved immunity, antioxidation and optimized bacterial composition, but has no significant effect on live weight gain or abundance of potentially pathogenic bacteria^46^. Furthermore, the effect of SEBS was greater than that of IS in this experiment, suggesting that Se availability was greater with SEBS supplementation. Therefore, SEBS combined the biological activities of Se and *B. subtilis yb-1*14,246.

In conclusion, our study reported the colonization of probiotic bacteria *B. subtilis yb-1*14,246 in distal ileal mucous membrane using FISH and qRT-PCR. Thereafter, we observed that the composition of intestinal microbiota and immunity were improved under the action of colonization. Se binding to the body of *B. subtilis yb-1*14,246 can more promote body growth and immunity, and the combined use of Se and *B. subtilis yb-1*14,246 as SEBS induced further improvements compared to those observed of each one administered alone. Overall, SEBS improved body growth, immune and decreased mortality, and our research provided a new avenue in use of probiotics and essential micro-elements.

## Data Availability

The sequencing data of cecal microbiota is deposited into the Sequence Read Archive database (SRP) of NCBI (SRR13290974). The BioProject accession number is PRJNA684959.
